# Chromosome-scale assembly of barley cv. ‘Haruna Nijo’ as a resource for barley genetics

**DOI:** 10.1093/dnares/dsac001

**Published:** 2022-01-12

**Authors:** Areej Sakkour, Martin Mascher, Axel Himmelbach, Georg Haberer, Thomas Lux, Manuel Spannagl, Nils Stein, Shoko Kawamoto, Kazuhiro Sato

**Affiliations:** 1 Institute of Plant Science and Resources, Okayama University, Kurashiki 710-0046, Japan; 2 Department Genebank, Leibniz Institute of Plant Genetics and Crop Plant Research (IPK), OT Gatersleben, 06466 Seeland, Germany; 3 German Centre for Integrative Biodiversity Research (iDiv) Halle-Jena-Leipzig, 04103 Leipzig, Germany; 4 Plant Genome and Systems Biology (PGSB), Helmholtz Center Munich, German Research Center for Environmental Health, 85764 Neuherberg, Germany; 5 Center for Integrated Breeding Research (CiBreed), Georg-August-University Göttingen, 37075 Göttingen, Germany; 6 Department of Informatics, National Institute of Genetics, Mishima 411-8540, Japan

**Keywords:** *Hordeum vulgare*, full-length cDNA, RNA-Seq, genome sequencing, pseudomolecules

## Abstract

Cultivated barley (*Hordeum vulgare* ssp. *vulgare*) is used for food, animal feed, and alcoholic beverages and is widely grown in temperate regions. Both barley and its wild progenitor (*H. vulgare* ssp. *spontaneum*) have large 5.1-Gb genomes. High-quality chromosome-scale assemblies for several representative barley genotypes, both wild and domesticated, have been constructed recently to populate the nascent barley pan-genome infrastructure. Here, we release a chromosome-scale assembly of the Japanese elite malting barley cultivar ‘Haruna Nijo’ using a similar methodology as in the barley pan-genome project. The 4.28-Gb assembly had a scaffold N50 size of 18.9 Mb. The assembly showed high collinearity with the barley reference genome ‘Morex’ cultivar, with some inversions. The pseudomolecule assembly was characterized using transcript evidence of gene projection derived from the reference genome and *de novo* gene annotation achieved using published full-length cDNA sequences and RNA-Seq data for ‘Haruna Nijo’. We found good concordance between our whole-genome assembly and the publicly available BAC clone sequence of ‘Haruna Nijo’. Interesting phenotypes have since been identified in Haruna Nijo; its genome sequence assembly will facilitate the identification of the underlying genes.

## 1. Introduction

Cultivated barley is used for many purposes, including animal feed, human food, and malting for brewing. Malting barley has only been cultivated in Japan for ca. 140 years.^1^ The founder cultivars were mainly introduced from Europe and crossed with Japanese landraces, which prior to that had been used for human food. In 1978, the malting barley cultivar ‘Haruna Nijo’ was released from Sapporo Breweries (Tokyo, Japan) and has since been used as a donor of high-quality profiles in Japanese malting barley breeding programs.

At Okayama University (Okayama, Japan), ‘Haruna Nijo’ is used as a key genotype in genetics and genomics studies. ‘Haruna Nijo’ was used for the generation of expressed sequence tags (see also https://harvest.ucr.edu/ last accessed Jan 19, 2022).^2^ Using these transcript sequences, a high-density genetic map was constructed from a cross between ‘Haruna Nijo’ and the wild barley (*H. vulgare* ssp. *spontaneum*) accession ‘OUH602’,^3^^,^^4^ and a set of recombinant chromosome substitution lines was developed.^5^ ‘Haruna Nijo’ was used to generate full-length cDNA (fl-cDNA) sequences,^6^^,^^7^ which have been used for the annotation of gene models in the reference genome of the cultivar ‘Morex’.^8–11^ Whole-genome shotgun sequencing was performed for ‘Haruna Nijo’ to enable the estimation of the genic regions of the genome.^8^^,^^12^ A BAC library of ‘Haruna Nijo’ was also constructed to isolate the genes responsible for major traits,^13^ such as hull-less caryopsis^14^ and seed dormancy.^15^ The mitochondrial genome of ‘Haruna Nijo’ was also sequenced and found to be highly similar to that of ‘OUH602’.^16^

After the release of a high-quality barley genome assembly generated using BAC-by-BAC sequencing and scaffold alignment,^9^ several whole-genome shotgun assembly techniques were developed for Illumina short reads, such as the DeNovoMAGIC assembly pipeline (NRGene, Ness Ziona, Israel), the TRITEX pipeline,^17^ and w2rap-contigger.^18^ Using these assembly methodologies, the global landscape of the barley genome (pan-genome)^19^ was recently analysed using 20 domesticated and wild accessions^10^ based on a selection of 22,000 genomic profiling datasets (GBS) from German gene bank accessions.^20^

Here, we utilized the TRITEX pipeline to generate a chromosome-scale genome assembly of ‘Haruna Nijo’. We aligned the assembly to the most recently updated assembly, ‘Morex’V3,^11^ to identify genomic differences among the genotypes. We also aligned the assembly to the published BAC sequences used for gene isolation to estimate the quality of the assembly. A similar sequencing methodology was also recently applied to the wild barley accession ‘OUH602’^21^; however, the assembly of the ‘Haruna Nijo’ genome is desirable for its economic and breeding importance.

The present barley genome annotation, e.g. EnsemblPlants (http://plants.ensembl.org/Hordeum_vulgare/ last accessed Jan 19, 2022), is based on ‘Morex’, which is the North American malting cultivar with a Manchurian landrace pedigree, and differs from malting barleys in other areas of the world. In a recent barley pan-genome analysis,^10^ gene projection was performed using informant gene models of ‘Morex’, the German malting cultivar ‘Barke’, and an Ethiopian landrace ‘HOR10350’, which were predicted from transcriptome data and protein homology information using a previously described annotation pipeline.^9^ In addition to this gene projection analysis, we performed *de novo* gene annotation for ‘Haruna Nijo’ using published fl-cDNA sequences and RNA-Seq data. These procedures may provide alternative gene annotation information on the barley genome by characterizing different sources of transcript and protein information from fl-cDNA sequences and RNA-Seq data.

## 2. Materials and methods

### 2.1 DNA extraction, library construction, and sequencing

High-molecular-weight DNA was isolated from leaf material of seedlings of ‘Haruna Nijo’^22^ and size selected for a molecule size of 40 kb or higher. The 440-bp paired-end (PE) libraries were prepared with the Hyper Kapa Library Preparation kit (Kapa Biosystems) with no polymerase chain reaction amplification. The 8- to 10-kb mate-pair (MP) libraries were constructed with the Nextera Mate Pair library Sample Prep kit (Illumina, San Diego, CA, USA) followed by the TruSeq DNA Sample Prep kit. The 10X libraries were constructed with the Chromium Genome Library Kit & Gel Bead Kit v2 (10X Genomics). Sequencing was performed following Sato et al.^21^ In brief, the 440-bp PE libraries were sequenced for 251 cycles using a NovaSeq 6000 system (Illumina). The 10X and MP libraries were sequenced for 151 cycles from each end of the fragments on the NovaSeq 6000 system. All libraries were prepared and sequenced at the University of Illinois Roy J. Carver Biotechnology Center (Urbana, IL, USA). *In situ* Hi-C libraries were prepared as described by Padmarasu et al.^23^ Sequencing data generated from each of the libraries are listed in Supplementary Table S1. The Hi-C data were used to prepare chromosome-scale assemblies using the TRITEX pipeline,^19^ which was also used for the contig assembly and scaffolding with the PE, MP, and 10X data (Supplementary Table S1).

### 2.2 Transcript sequencing

Published RNA-Seq reads from the seedling root, shoot, spike at flowering, and seeds of ‘Haruna Nijo’^12^ were used for the transcript sequencing. An additional RNA sample of a young spike (3 cm in length) from ‘Haruna Nijo’ was also extracted and subjected to an RNA-Seq analysis, as described by Sato et al.^12^ These RNA-Seq libraries were sequenced with the MiSeq Reagent Kit V3 (2 × 300 bp cycles) on a MiSeq system (Illumina).

### 2.3 Gene projection

To derive the projected gene structures for ‘Haruna Nijo’, informant gene models of ‘Morex’, ‘Barke’, and ‘HOR10350’ were employed, which were predicted from transcriptome data and protein homology information^10^ using a previously described annotation pipeline.^9^ The projection was based on a stepwise procedure, as previously described.^10^^,^^21^ Briefly, BLASTN^24^ and Exonerate alignments^25^ of the coding sequences (CDSs) of each of the barley sources of the ‘Haruna Nijo’ genome sequence were computed. The matches were clustered by their genomic loci, and the top-scoring match was selected using a stepwise integration approach. In addition to protein-coding genes, ‘pseudogene’-type mappings were previously projected and included in the CDSs and GFF files but were obviously missing from the protein sequence files.

### 2.4 *De novo* gene annotation using RNA-Seq and fl-cDNA sequences

A structural gene annotation was performed by combining *de novo* gene calling and homology-based approaches with RNA-Seq, protein, isoseq, and fl-cDNA datasets. Using evidence derived from expression data, RNA-Seq sequences were first mapped against the ‘Haruna Nijo’ genome assembly using STAR^26^ (version 2.7.8a) and subsequently assembled into transcripts using StringTie^27^ (version 2.1.5; parameters -m 150-t-f 0.3). Triticeae protein sequences obtained from publicly available datasets (UniProt; https://www.uniprot.org; last accessed Jan 19, 2022 accessed 10 December 2021) were aligned against the genome sequence using GenomeThreader^28^ (version 1.7.1; arguments -startcodon-finalstopcodon -species rice -gcmincoverage 70 -prseedlength 7 -prhdist 4). The fl-cDNAs and isoseq were aligned to the genome assembly using GMAP^29^ (version 2018-07-04). All RNA-Seq, fl-cDNA, and aligned protein sequences were combined using Cuffcompare^30^ (version 2.2.1) and subsequently merged with StringTie (version 2.1.5; parameters –merge -m150) into a pool of candidate transcripts. TransDecoder (version 5.5.0; http://transdecoder.github.io last accessed Jan 19, 2022) was used to find potential open reading frames (ORFs) and to predict protein sequences within the candidate transcript set. An *ab initio* annotation was performed using Augustus^31^ (version 3.3.3). GeneMark^32^ (version 4.35) was additionally used to further improve the structural gene annotation. To avoid potential over-prediction, guiding hints were generated using the above-described RNA-Seq, isoseq, protein, and fl-cDNA datasets and were then trained and optimized using a specific Augustus model for barley, as described by Hoff and Stanke.^31^ Structural gene annotations from different prediction methods were combined using EVidenceModeler^33^ (version 1.1.1), and the weights were adjusted according to the input source: *ab initio* (Augustus: 5, GeneMark: 2) and homology based (10). Additionally, two rounds of PASA^34^ (version 2.4.1) were run to identify untranslated regions and isoforms using the above-described fl-cDNA dataset.

BLASTP^24^ (ncbi-blast-2.3.0+, parameters -max_target_seqs 1 -evalue 1e-05) was used to compare potential protein sequences with a trusted set of reference proteins (Uniprot Magnoliophyta, reviewed/Swissprot; downloaded on 3 August 2016; https://www.uniprot.org last accessed Jan 19, 2022). This differentiated candidates into complete and valid genes, non-coding transcripts, pseudogenes, and transposable elements. In addition, the PTREP database (Release 19; http://botserv2.uzh.ch/kelldata/trep-db/index.html last accessed Jan 19, 2022) was used in the BLASTP analysis; this database of hypothetical proteins contains deduced amino acid sequences in which internal frameshifts have been removed in many cases. This step is particularly useful for the identification of divergent transposable elements with no significant similarity at the DNA level. The best hits were selected for each predicted protein to each of the three databases: UniProt, SwissProt, and PTREP. Only hits with an e-value below 10^e-10^ were considered. Furthermore, the functional annotation of all predicted protein sequences was performed using the AHRD pipeline (https://github.com/groupschoof/AHRD last accessed Jan 19, 2022).

The proteins were further classified into two confidence classes: high and low. Hits with subject coverage (for protein references) or query coverage (transposon database) above 80% were considered significant. The proteins were classified as high confidence if the sequence was complete and had a subject and query coverage above the threshold in the UniMag database or no BLAST hit in UniMag or PTREP but present in UniPoa. A protein sequence was defined as being low confidence if it was incomplete and had a hit in the UniMag or UniPoa database but not in PTREP. Alternatively, complete protein sequences with no hit in UniMag, UniPoa, or PTREP were also classified as low confidence. In a second refinement step, low-confidence proteins with an AHRD-score of 3* were promoted to high-confidence.

### 2.5 Repeat and transcript annotation

The final assembly was analysed for repetitive regions using RepeatMasker^35^ (version 4.0.9) with the TREP repeat library^36^ (trep-db_complete_Rel-19; downloaded from http://botserv2.uzh.ch/kelldata/trep-db/downloadFiles.html last accessed Jan 19, 2022 on 13 September 2020). The repetitive regions were changed to lowercase (-xsmall parameter). The output of RepeatMask was condensed using the perl script ‘one-code-to-find-them-all’^37^ with the parameters -strict and -unknown.

### 2.6 Data validation and quality control

Benchmarking Universal Single-Copy Orthologs^38^ (BUSCO; version 3.0.2) was used with the plant dataset (embryophyta_odb10) to validate the assembly and gene models. For gene prediction, BUSCO uses Augustus^39^^,^^40^ (version 3.3). For the gene-finding parameters in Augustus, the species was set to wheat and BUSCO was run in genome mode (-m geno -sp wheat).

### 2.7 Alignment of published BAC sequences

Published ‘Haruna Nijo’ BAC clone sequences of kernel row type *Vrs1*,^41^ brittle rachis *Btr1* and *Btr2*,^42^ and quantitative locus seed dormancy 1 *Qsd1*^15^ were downloaded from NCBI. Each clone sequence was aligned to the pseudomolecule sequences of ‘Haruna Nijo’ and ‘Morex’V3 using minimap2.^43^

### 2.8 Genome browser

Pseudomolecule assembly, gene models of the CDS, and amino acid models were visualized in Jbrowse genome browser (version 1.16.9). The BLAST (version 2.2.18) and BLAT (version 34) servers were also installed to search for target sequences in the pseudomolecules and gene models.

### 2.9 Data availability

Raw reads have been deposited in the ENA sequence read archive. Bioproject: PRJEB44504 (ERS_ID: paired-end reads: ERS6294308; mate-pair reads: ERS6294309; 10X reads: ERS6294307; Hi-C reads: ERS6294313; assembly: ERS6294316) (Supplementary Table S2).

The reference assembly is available for download or BLAST search from http://viewer.shigen.info/harunanijo/index.php. last accessed Jan 19, 2022

## 3. Results and discussion

### 3.1 Genome assembly

**Figure 1:**
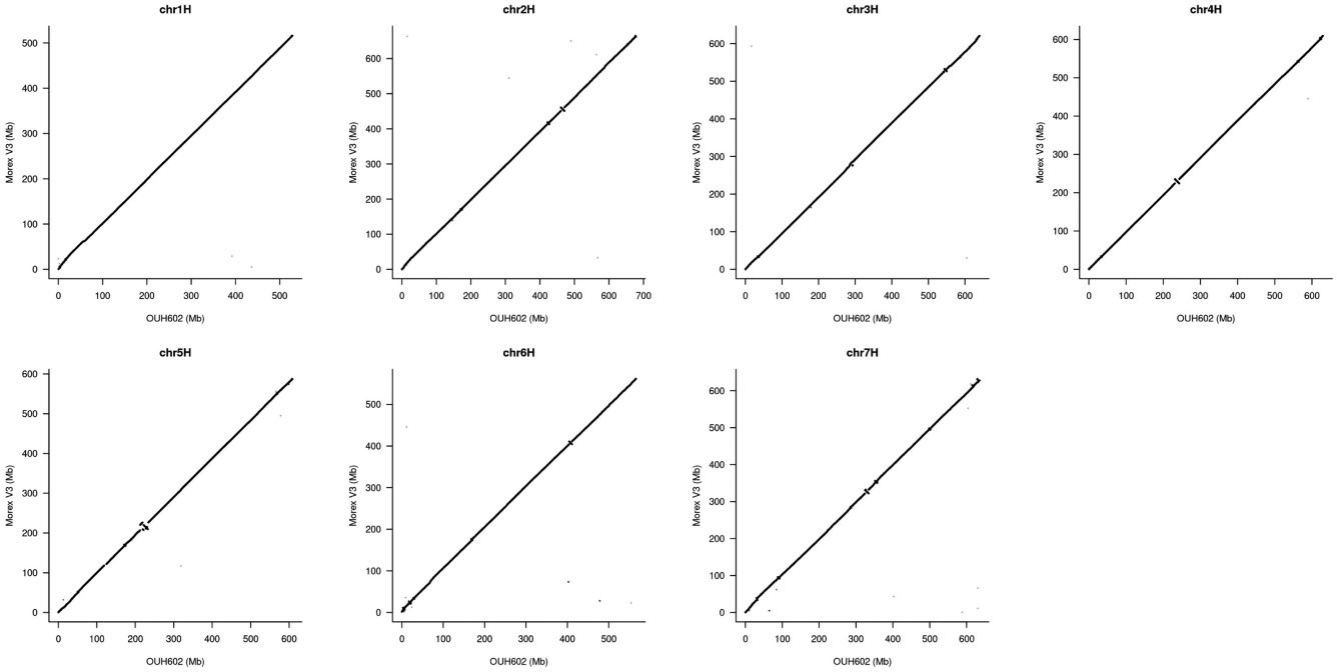
Alignment of pseudomolecules of ‘Haruna Nijo’ to ‘Morex’V3 individual chromosomes.

We generated the genome assembly from PE and MP short reads and 10X reads. Approximately 868 Gb of raw data was generated, providing an estimated 170× coverage of the genome (Supplementary Table S1). An assembly generated using the TRITEX pipeline^17^ resulted in a scaffold N50 value of 18.9 Mb (Table 1). We integrated Hi-C data into the assembly, which uses a genomic distance matrix inferred from native chromatin folding to increase the scaffold-level contiguity to full chromosome size (Supplementary Fig. S1). The final pseudomolecule size was 4.28 Gb, comprising 552 scaffolds and a cumulative size of unanchored scaffolds of 154.3 Mb. The pseudomolecule size of ‘Haruna Nijo’ is comparable with that of the pan-genome assemblies of ‘Morex’V2 obtained using similar sequencing platforms but with a smaller scaffold N50 value. The datasets for ‘Morex’V3 showed improved statistics compared with our assemblies due to the use of accurate long-read sequencing by circular consensus sequencing on the PacBio platform in the generation of this assembly.^11^ The alignment of the pseudomolecules of ‘Haruna Nijo’ to ‘Morex’V3 individual chromosomes revealed some small inversions (Fig. 1); however, the overall contiguity of entire chromosomes was retained between ‘Haruna Nijo’ and ‘Morex’V3.

**Table 1:** Statistics of ‘Haruna Nijo’ and two versions of ‘Morex’ assemblies

Parameter	‘Haruna Nijo’	‘Morex’V2	‘Morex’V3
Number of scaffolds in pseudomolecules	552	273	103
Pseudomolecule size (Gb)	4.28	4.34	4.20
Scaffold N50^a^ [Mb]	18.9	43.7	118.9
Scaffold N90 [Mb]	2.6	5.9	21.8
Cumulative size of unanchored scaffold (Mb)	154.3	82.9	29.1

a ‘Scaffold’ refers to top-level entities that constitute the pseudomolecules. In ‘Morex’V3, these are Bionano scaffolds of PacBio HiFi contigs; in the other assemblies, superscaffolds were constructed from PE, MP, and 10X data.

For easy access, the reference sequence is available in BLAST-searchable form at http://viewer.shigen.info/harunanijo/index.php last accessed Jan 19, 2022.

### 3.2 Quality of assemblies

We used the spectra-cn function from the Kmer Analysis Toolkit (KAT)^44^ to compare *k*-mer contents in the scaffolds and pseudomolecules. KAT generates a *k*-mer frequency distribution from the PE and MP reads and identifies how many times *k*-mers from each part of the distribution appear in the assemblies being compared.^21^ The spectra-cn plot in Supplementary Fig. S2 generated from the contigs shows sequencing errors (*k*-mer multiplicity <20) in black, as these are not included in the assembly. Most of the content appears in a single red peak, indicating sequences that appear once in the assembly. The black region under the main peak is small, indicating that most of this content from the reads is present in the assembly. The content that appears to the right of the main peak and is present two or three times in the assembly represents repeats. Pseudomolecules may contain more miss-assemblies than scaffolds; this is not obvious in the spectra-cn plot in Supplementary Fig. S2b.

We evaluated the quality of the ‘Haruna Nijo’ assembly using BUSCO.^38^^,^^45^ This program assesses the completeness of a genome by identifying conserved single-copy orthologous genes. The scaffold and pseudomolecule stages had complete single-copy genes at a rate of 96.0% and 95.7%, respectively (Table 2). These values are very close to those recently published for the ‘Morex’V2 assembly, which had 97.2% single-copy genes.^46^ The differences are mainly due to the greater number of duplicated genes in the scaffolds (1.3%) than the pseudomolecules (1.2%). Only 1.0% of the fragmented sequences were present in both the scaffolds and pseudomolecules.

**Table 2:** BUSCO statistics of ‘Haruna Nijo’

Factor	Scaffolds	Pseudomolecule
Complete BUSCOs	1,403 (97.5%)	1,396 (96.9%)
Complete BUSCOs: single copy	1,382 (96.0%)	1,378 (95.7%)
Complete BUSCOS: duplicated	21 (1.3%)	18 (1.2%)
Fragmented BUSCOs	14 (1.0%)	14 (1.0%)
Missing BUSCOs	23 (1.5%)	30 (2.1%)
Total BUSCO groups searched	1,440	1,440

### 3.3 Repeat masking

We analysed each chromosome of the ‘Haruna Nijo’ assembly for repetitive regions using RepeatMasker with the TREP repeat library. This analysis identified 72.8% (3.23 Gb) of the ‘Haruna Nijo’ assembly as transposable elements (Supplementary Table S3), almost all of which were retroelements. The same analysis was performed for ‘Morex’V2 and ‘Morex’V3, producing similar results (Supplementary Table S3). The differences from the published results for the ‘Morex’V2 and ‘Morex’V3 assemblies^11^^,^^17^ were due to the different repeat libraries used.

### 3.4 Gene projection

We assessed the gene content of ‘Haruna Nijo’ using a gene projection approach, as described by Jayakodi et al.^10^ for the 20 barley pan-genome assemblies. The total number of loci was 47,367, which is within the range of 42,464 to 47,588 reported for the 20 pan-genome assemblies. Of the 44,579 protein-coding genes, between 42,800 and 43,211 loci had a BLAST match with an e-value of <1–30, and 34,427 and 38,005 were one-to-one reciprocal BLAST orthologs between ‘Haruna Nijo’ and ‘B1K-04-12’ or ‘Morex’V2, respectively. The overall and orthologous gene content of ‘Haruna Nijo’ is therefore highly conserved in comparison with other barley lines. Likewise, 15.9% (7,109) of the tandem-repeated genes in ‘Haruna Nijo’ had similar ranges as were detected for the 20 barley pan-genome assemblies and were located in 2,735 clusters. The gene content statistics above indicate that the ‘Haruna Nijo’ assembly contains a gene set with highly similar characteristics to those reported for the 20 barley pan-genome assemblies.

### 3.5 *De novo* gene annotation using RNA-Seq and fl-cDNA sequences

**Figure 2:**
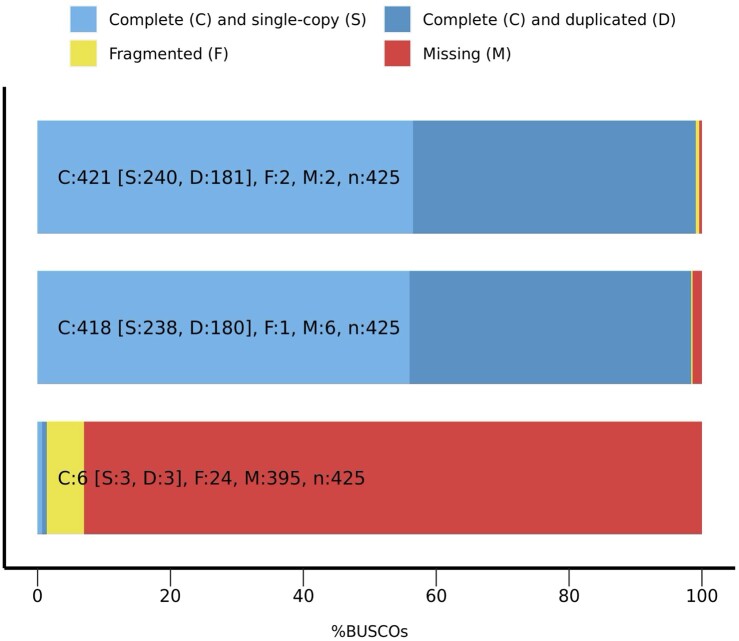
BUSCO assessment results of ‘Haruna Nijo’ fl-cDNA sequences (upper), high-confidence genes (middle), and low-confidence genes (lower).

A final structural gene annotation yielded 161,721 gene models, including 49,524 high- and 112,197 low-confidence gene models (Table 3). The high number of total gene models is likely due to the *ab initio* prediction step, which was run without the use of transposable elements hints; the high number of low-confidence gene models supports this rationale. The BUSCO score of the high-confidence genes was 98.4 (Fig. 2). The average number of transcripts per gene was 1.39 for the high-confidence gene models, which was much higher than 1.01 for the low-confidence gene models.

**Table 3:** *De novo* gene annotation statistics

Statistics	Complete sequences	High confidence	Low confidence
Number of genes	161,721	49,524	112,197
Number of monoexonic genes	67,724	12,645	55,079
Number of transcripts	181,980	68,751	113,229
Transcripts per gene	1.13	1.39	1.01
cDNA lengths (mRNAs)	1,294	1,696	1,050
CDS lengths (mRNAs)	1,154	1,377	1,018
Exons per transcript (mRNAs)	3.45	5.21	2.38
Exon lengths (mRNAs)	375	326	441
Intron lengths (mRNAs)	675	623	770
CDS exons per transcript (mRNAs)	3.33	4.95	2.35
CDS exon lengths	346	278	434
5' UTR exon number	54,193	48,584	5,609
3' UTR exon number	52,989	44,690	8,299

We next compared our sequences with the fl-cDNA dataset, which consisted of 22,651 sequences generated from ‘Haruna Nijo’.^6^^,^^7^ These sequences were created from plants grown in 12 different conditions and thus represent a good snapshot of the barley transcriptome. The average insert size of these fl-cDNA sequences was 1,711 bp, which was close to the cDNA length of the high-confidence gene models. Sequence similarities among our data and the fl-cDNA sequences, gene models of gene projection, and *de novo* gene annotations were compared using a BLASTN analysis with a threshold e-value of <−100 (Table 3). The 22,651 fl-cDNA query sequences showed high similarity with the sequences from the gene projection (19,771) and *de novo* annotation (19,636) (Table 4). These numbers are consistent with the number of fl-cDNA sequences with complete ORFs (19,335) reported by Matsumoto et al.^7^; other fl-cDNA sequences had truncated ORF or non-protein-coding sequences. The results also indicated that almost 10% of each gene model did not overlap each other. The amino acid sequences showed a lower level of overlapping than the nucleotide sequences (0.707–0.731; Supplementary Table S4).

**Table 4:** BLASTN hits (<e-100) among nucleotide sequences of fl-cDNA, gene projection, and *de novo* gene model sequences

Target	Query		
	Full-length cDNA	Gene projection	*De novo* annotation
Full-length cDNA	22,651	25,977	28,415
Gene projection	19,711	47,367	43,087
*De novo* annotation	19,636	42,336	49,524
Total hits	19,937	42,753	44,387
Ratio (total hits/number of queries)	0.880	0.903	0.896

### 3.6 Alignment with BAC clone sequences

**Figure 3:**
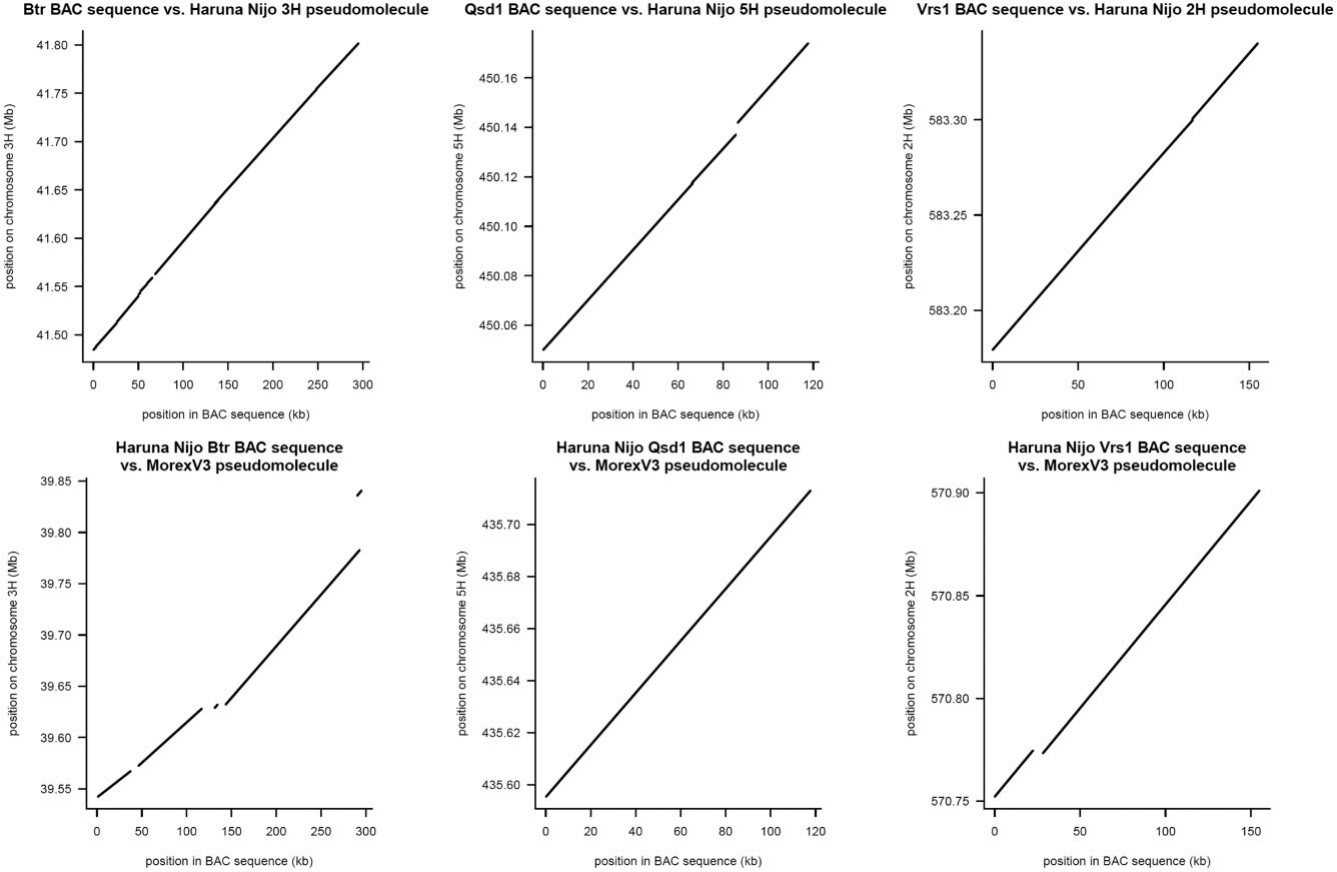
Alignment of ‘Haruna Nijo’ BAC sequences of *Btr*, *Qsd1*, and *Vrs1* regions to pseudomolecules of ‘Haruna Nijo’ and ‘Morex’V3.

We aligned ‘Haruna Nijo’ BAC clone sequences to pseudomolecules of ‘Haruna Nijo’ to estimate the contiguity of both sequences (Fig. 3). The BAC clones were analysed using shotgun Sanger sequencing and assembled on an individual clone basis. The BAC clone sequences of *Btr1*/*Btr2* were composed of several clones and showed apparent discontinuity with the pseudomolecule sequence of ‘Haruna Nijo’. The alignment of these BAC sequences with the ‘Morex’V3 pseudomolecule sequence revealed fragmentation at the 3' region, but the 5' region showed higher contiguity. Another BAC clone sequence, *Qsd1*, which was derived from a single clone, showed more contiguity with the pseudomolecules of ‘Haruna Nijo’; however, there was a significant gap between the BAC sequence and the pseudomolecule sequence of ‘Morex’V3. The quality of the BAC sequences was comparable with that of ‘Morex’V3, but with some structural disorders.

The observed mismatches between BAC clones and pseudomolecules indicate that the pseudomolecules of ‘Haruna Nijo’ do not have as high of a sequencing quality as those of ‘Morex’V3; however, they are useful for examining contiguity in the genome for gene identification.

### 3.7 Genome browser

The high-performance and user-friendly graphical interface genome browser Jbrowse was used to visualize the pseudomolecule sequence and the gene models. Tracks of *de novo* annotations and gene projections each display the result of the associated annotation (e.g. exon structure, protein names, and transposable elements) to allow a comparison of each gene model. The fl-cDNA sequence track based on the BLAST search result against the pseudomolecule sequence was also provided, showing strict similarity to clones only. In addition to the browser, the user interface of the sequence similarity search programs BLAST and BLAT was also provided. The BLAST search results are directly linked to Jbrowse as a user track, which allows the mapping of query sequences against the reference genome and their comparison with the gene models. The assembled sequence and annotation files can be downloaded from the website (http://viewer.shigen.info/harunanijo/index.php last accessed Jan 19, 2022) so that our data can be used in the local user’s environment.

### 3.8 Conclusion

Here, we present an assembly of ‘Haruna Nijo’ that is of similar quality to the ‘Morex’V2 reference.^17^ Importantly, it is a European-style Japanese two-row cultivar, expanding barley genomic resources to Japanese and European breeding materials in contrast to the American six-row cultivar ‘Morex’. Interesting phenotypes have since been identified in Haruna Nijo; its genome sequence assembly will facilitate the identification of the underlying genes.

## Supplementary data

Supplementary data are available at DNARES online.

## Supplementary Material

dsac001_Supplementary_Data
