# Supplementary material for: Chromosome-scale assembly of barley cv. ‘Haruna Nijo’ as a resource for barley genetics
Source: DNA Res. 2022 Jan 12;29(1):dsac001. doi: 10.1093/dnares/dsac001 (PMC8798153; doi:10.1093/dnares/dsac001)
Supplement: dsac001_Supplementary_Data [file dsac001_supplementary_data.zip › Supplementary Figs.pdf]

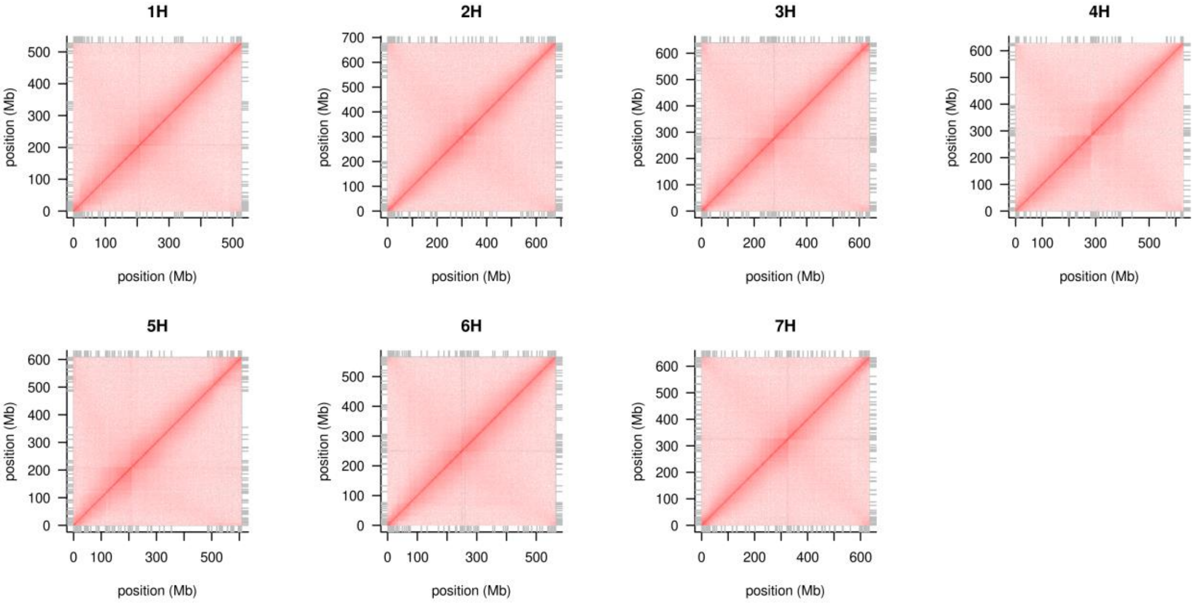

Supplementary Fig. 1. Intra-chromosomal Hi-C contact matrices. Gray lines mark contig boundaries. Some centromeres are spanned by a single contig. The absence of off-(anti)-diagonal signals supports the accuracy of the assembly.

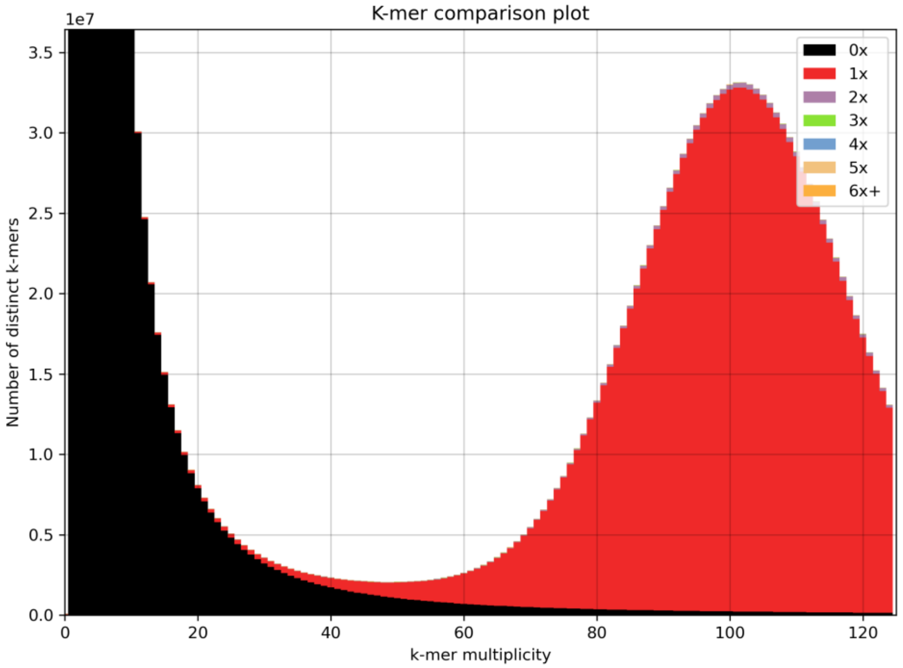

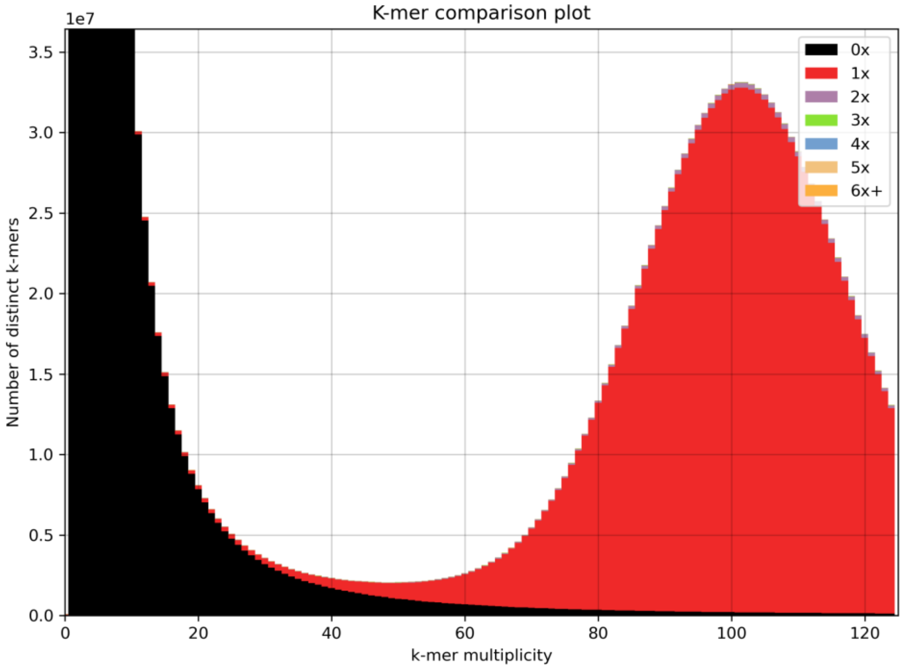

Supplementary Fig. 2. Spectra cn plots comparing *k*-mers from the paired end and mate pair reads to *k*-mers in pseudomolecule assembly (upper) and scaffold assembly (lower).
